# Successful Infection of Domestic Pigs by Ingestion of the European Soft Tick *O. Erraticus* That Fed on African Swine Fever Virus Infected Pig

**DOI:** 10.3390/v12030300

**Published:** 2020-03-11

**Authors:** Rémi Pereira De Oliveira, Evelyne Hutet, Maxime Duhayon, Jean-Marie Guionnet, Frédéric Paboeuf, Laurence Vial, Marie-Frédérique Le Potier

**Affiliations:** 1 UMR ASTRE, Centre de Coopération Internationale en Recherche Agronomique pour le Développement (CIRAD), F-34398 Montpellier, France; remi.pereira_de_oliveira@cirad.fr (R.P.D.O.); maxime.duhayon@cirad.fr (M.D.); laurence.vial@cirad.fr (L.V.); 2 UMR ASTRE, Univ Montpellier, Centre de Coopération Internationale en Recherche Agronomique pour le Développement (CIRAD), Institut National de Recherche pour l’Agriculture, l’Alimentation et l’Environnement (INRAE), F-34398 Montpellier, France; 3 Unité de Virologie et Immunologie Porcines, Laboratoire de Ploufragan-Plouzané-Niort, Agence Nationale de Sécurité Sanitaire (ANSES), 22440 Ploufragan, France; evelyne.hutet@anses.fr (E.H.); jean-marie.guionnet@anses.fr (J.-M.G.); frederic.paboeuf@anses.fr (F.P.)

**Keywords:** African swine fever, domestic pig, transmission, ingestion, soft tick, *Ornithodoros erraticuss*

## Abstract

African swine fever is a highly lethal hemorrhagic fever of *Suidae*, threatening pig production globally. *Suidae* can be infected by different ways like ingestion of contaminated feed, direct contact with infected animals or fomites, and biting by infected soft tick bites. As already described, European soft ticks (*Ornithodoros erraticus* and *Ornithodoros verrucosus*) were not able to transmit African swine fever virus by biting pigs although these ticks maintained the infectious virus during several months; therefore, the possibility for pigs to become infected through the ingestion of infected ticks was questioned but not already explored. To determine if such oral ingestion is an alternative pathway of transmission, *O. erraticus* ticks were infected by blood-feeding on a viremic pig infected with the European African swine fever virus strain Georgia2007/1, then frozen at zero and two months post-engorgement, then after, were embedded in the food to pigs. Pig infection was successful, with superior efficiency with ticks frozen just after the infectious blood meal. These results confirmed the potential role of *O. erraticus* ticks as an ASFV reservoir and demonstrated the efficiency of non-conventional pathways of transmission.

## 1. Introduction

African swine fever (ASF) is a hemorrhagic fever disease of domestic pigs and wild boar caused by the African swine fever virus (ASFV). ASFV was re-introduced in Europe in 2007 in Georgia and has further spread to Europe and Asia [1]. This spread is partly due to direct contact between pigs and wild boar, but also to human activities such as importation/transportation of infected animals or contaminated feed and fomites. Another possible way of spread is a vectorial transmission. Although ASFV can be transmitted by some species of *Ornithodoros* soft ticks in endemic African areas, Pereira de Oliveira (2019) experimentally showed that Palearctic soft ticks, *O. erraticus* (from Portugal) and *O. verrucosus* (from Ukraine), were not able to transmit the current circulating European ASFV strain Georgia2007/1 [2]. However, this study demonstrated that the virus was still infectious up to eight months in *O. erraticus* and two months in *O. verrucosus* [2], which raises questions about the possibility of transmission through other pathways than tick bites.

Among the different routes for introducing ASFV in new areas, including direct or indirect contacts with pig feces and urines [3,4,5], the importation of infected food has been described as an important pathway for virus spread in Russia [6]. ASFV was shown to be still infectious in different pig tissues, for example, in muscles collected from abattoir during epizooties in Spain during 1960–1990 [7]. Furthermore, the virus was still detected in the muscle tissue for about 98 to 112 days after industrial processing [7]. ASFV was also isolated from dried salami and pepperoni sausages but not after the curing period [8]. More recently, Italian dry-cured meat products from pigs experimentally infected with the current Sardinian ASFV strain were able to infect naïve pigs [9]. In addition, feed and feed ingredients could stay contaminated for a few weeks with the current Eurasian ASFV strain Georgia2007/1 [10]. This makes infection by ingestion one of the most important routes of virus transmission. 

Recently, pig infection due to the ingestion of ASFV from stable flies fed on blood from ASFV-infected pigs and leeches’ blood-fed on ASFV-infected pigs was described [11,12]. Furthermore, stable flies were able to keep infectious ASFV up to two days [13], and ASFV was able to persist up to 160 days in leeches [12]. Since ASF has been re-introduced in the European Union, a seasonal pattern of outbreaks has been reported with more outbreaks in domestic pig herds in summer, the activity period for many dipteran species. One of the hypotheses to explain this epidemiological situation could be related to arthropod vectors, and more widely hematophagous species that could serve in the spread and the persistence of ASFV in infected areas.

Taking into account the foraging habits of *Suidae* [14,15] and the endophilic character of *Ornithodoros* [16] ticks, our study assesses the possibility of pigs to become infected by ingestion of infected ticks and to evaluate the risks of ASF persistence in Europe.

## 2. Materials and Methods 

### 2.1. Virus and Ticks

The Georgia2007/1 strain used in this study is a highly virulent ASFV strain. It was isolated from a domestic pig originating from Georgia [17], and kindly provided by Dr. Linda Dixon (OIE reference laboratory, Pirbright Institute, UK). This strain was amplified on porcine alveolar macrophages twice before being intramuscularly inoculated into pigs, as previously described [2]. First, three pigs were infected by intramuscular inoculation of the Georgia2007/1 ASFV strain using a dose of 10^3^ hemadsorbing dose 50% (HAD_50_) [18]. Pigs became viremic four days after inoculation. The infected pigs were used to infect ticks through natural blood-feeding when their viremia reached 10^7.8^ HAD_50_/mL.

The *Ornithodoros* soft tick species used in this study was *O. erraticus* from the Alentejo region in Portugal (named “Aletenjo” strain, collected from the field in 2013 and 2016 and reared in the insectary of CIRAD Montpellier with 1–5 generations completed since sampling). Only male and female adults were used in this study. Ticks were maintained in the laboratory at 26 °C with 80 to 90% relative humidity as recommended for this species [16]. After their infectious blood meal on viremic pigs, the ticks were frozen at −80 °C at zero (OeG-0) and two (OeG-2) months post-infection (PI) until their use for the transmission trial.

### 2.2. Viral Titration

In order to determine the viral titer in ticks consumed by pigs, viral titration was carried out on a set of ticks before the transmission trial. Ten (five males and five females) OeG-0 ticks and ten (same partitioning) OeG-2 ticks were analyzed. Soft ticks fed on ASFV-infected pig were crushed in 1 mL of sterile phosphate-buffered saline solution (PBS) using a Star–Beater (VWR) with one bead of 3 mm and one bead of 4 mm at 25 Hz during 3 min. Homogenate was centrifuged at 5000× *g* for 2 min and the virus titration was performed on the supernatant by the hemadsorption assay, and results were expressed in HAD_50_/mL [18].

### 2.3. Transmission Trial

Experiments were conducted in protected facilities at ANSES–Ploufragan, on 14 Large White, Specific Pathogen Free (SPF) piglets of 7 weeks old. 

Eleven pigs were split in four independent rooms. Fifty ticks were used for the transmission trial (25 males and 25 females). Two pigs were fed with OeG-0 (group 1). Three pigs were fed with OeG-2 ticks (group 2). Group 1 had a contact pig that received no treatment. Ticks were embedded in a piece of *brioche* to facilitate ingestion by pigs. Each pig ate five males and five females. The third group of three pigs ate a piece of *brioche* spiked with 10^5.5^ HAD_50_ of virus culture diluted in PBS, as control of infection through ingestion (group 3). Two negative control pigs ate the same quantity of *brioche* without any treatment (group 4). Each group of pigs was visited daily, and their rectal temperature was recorded for clinical signs, as previously described [19]. Blood (ethylene diamine tetraacetic acide-treated and dry tubes) was collected twice a week and on the first day of hyperthermia (temperature > 40.1 °C) and on the day of euthanasia.

At the end of the experiment (10 days post-ingestion), or at earlier stages, if animals became sick, pigs were humanely euthanized by an anesthetic overdose of Zoletil® at 5 mL per 50 kg of weight administered via the vena cava and then exsanguinated. The contact pig (pig #3, group 1) was euthanized on day 5 for ethical reasons, as a pig cannot be let alone.

### 2.4. Pig Diagnosis

Pigs were diagnosed by real-time Polymerase Chain Reaction (PCR) as previously described [20] to confirm if they were infected or not by ASFV. Briefly, a real-time PCR was performed on DNA extracted from EDTA stabilized blood. Serums, collected at the day of euthanasia from pigs exposed to ASFV but that remained healthy, as well as from negative control pigs, were analyzed by ELISA (ID Screen® African Swine Fever Indirect, ID Vet, France) to determine if they seroconverted. The ELISA test used permitted the detection of IgG. 

### 2.5. Ethics Statement

Animal experiments were authorized by the French Ministry for Research (project N° 2017062615498464) and approved by the national ethics committee ANSES/ENVA/UPEC (authorization N° 11/07/17-3).

## 3. Results and Discussion

**Figure 1:**
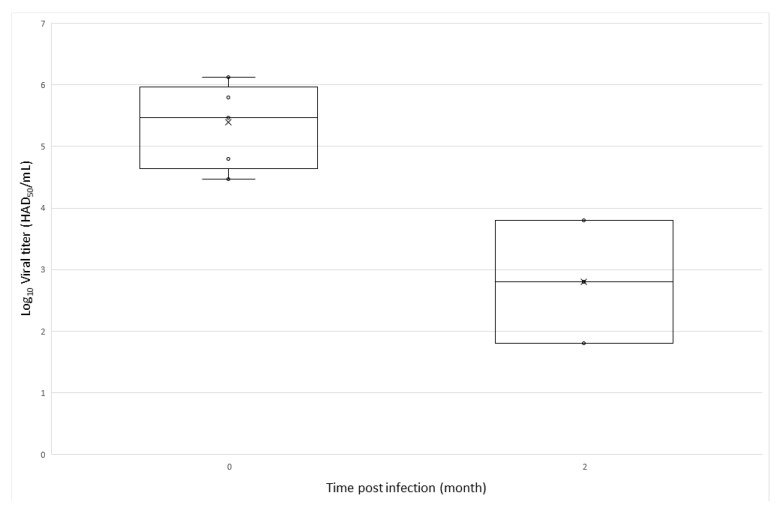
African swine fever viral titer of the soft ticks frozen at 0 and 2 months post infectious blood meal (10 ticks/group).

Virus was isolated from 9/10 and 7/10 OeG-0 and OeG-2 ticks, respectively. Viral titers of positive ticks were higher for OeG-0 ticks (viral titer > 10^4.4^ HAD_50_/mL) than OeG-2 ticks (viral titer < 10^4^ HAD_50_/mL) (Figure 1). Therefore, we estimated that pigs that had eaten OeG-0 and OeG-2 ticks received an average viral dose of 10^6^–10^7^ HAD_50_ and 10^4^–10^5^ HAD_50_, respectively.

Both pigs from group 1 that had eaten OeG-0 ticks became hyperthermic three to four days after exposure and were positive to ASFV diagnosis (Table 1). Only one out of the three pigs from group 2 that had eaten OeG-2 ticks became infected (Table 1). Against all expectations, the three control pigs from group 3 that received virus spiked *brioche* and the contact pig from group 1 did not develop any clinical signs and remained negative for ASFV. Negative controls pigs from group 4 never displayed clinical sign. No specific IgG antibodies to ASFV were detected in any of the pig sera tested.

These results confirm the possibility of infecting domestic pigs by the ingestion of ASFV-blood-fed soft ticks *O. erraticus*. Ticks with a high viral titer displayed the highest ability to infect the pigs through oral ingestion than ticks with a lower viral titer. The absence of infection with the “virus spiked *brioche*” was surprising as it was considered as a control of infection. This could be due to a low viral titer, too. Indeed, these results are in accordance with a previous study showing a better efficiency when viral titer is higher in contaminated food [21]. Moreover, the integrity of the virus when spiked in *brioche* might have been altered by salivary proteases, conversely to the virus present in the ticks. 

The fact that ticks remained sufficiently infectious two months post-infection to allow the infection of pigs by their ingestion confirms the possibility of *O. erraticus* to be a reservoir of ASFV strains circulating in Eurasia and suggest new transmission routes of ASFV by soft ticks *Ornithodoros* and more widely for others hematophagous organisms. Further studies are needed to better understand the role of the ingestion pathway in current epizooties in Eurasia, especially about the possible interactions between hematophagous species and wild boar.

## Figures and Tables

**Table 1:** Pig infection by ingestion of infected ticks at zero and two months post-infection or “virus spiked *brioche*”.

Pig	Pig Group	Initial Day of Hyperthermia	Day of Euthanasia	ASFV Diagnosis (PCR)	ASF Diagnosis (ELISA)
#1	1	3	4	+	NT
#2		4	5	+	NT
#3 (contact pig)		No hyperthermia	5	−	NT
#4	2	6	7	+	NT
#5		No hyperthermia	10	−	−
#6		No hyperthermia	10	−	−
#7	3	No hyperthermia	10	−	−
#8		No hyperthermia	10	−	−
#9		No hyperthermia	10	−	−
#10	4	No hyperthermia	10	−	−
#11		No hyperthermia	10	−	−

+: positive ASFV diagnosis; −: negative ASFV diagnosis. NT: not tested.

## References

[1] Chenais E., Depner K., Guberti V., Dietze K., Viltrop A., Ståhl K. (2019). Epidemiological considerations on African swine fever in Europe 2014–2018. Porc. Health Manag..

[2] Pereira de Oliveira R., Hutet E., Paboeuf F., Duhayon M., Boinas F., Perez de Leon A., Filatov S., Vial L., Le Potier M.-F. (2019). Comparative vector competence of the Afrotropical soft tick Ornithodoros moubata and Palearctic species, O. erraticus and O. verrucosus, for African swine fever virus strains circulating in Eurasia. PLoS ONE.

[3] Montgomery R.E. (1921). On A Form of Swine Fever Occurring in British East Africa (Kenya Colony). J. Comp. Pathol. Ther..

[4] Mazur-Panasiuk N., Żmudzki J., Woźniakowski G. (2019). African swine fever virus – persistence in different environmental conditions and the possibility of its indirect transmission. J. Vet. Res..

[5] Davies K., Goatley L.C., Guinat C., Netherton C.L., Gubbins S., Dixon L.K., Reis A.L. (2017). Survival of African Swine Fever Virus in Excretions from Pigs Experimentally Infected with the Georgia 2007/1 Isolate. Transbound. Emerg. Dis..

[6] Gogin A., Gerasimov V., Malogolovkin A., Kolbasov D. (2013). African swine fever in the North Caucasus region and the Russian Federation in years 2007–2012. Virus Res..

[7] Mebus C.A., House C., Gonzalvo F.R., Pineda J.M., Tapiador J., Pire J.J., Bergada J., Yedloutschnig R.J., Sahu S., Becerra V. (1993). Survival of foot-and-mouth disease, African swine fever, and hog cholera viruses in Spanish serrano cured hams and Iberian cured hams, shoulders and loins. Food Microbiol..

[8] McKercher P.D., Hess W.R., Hamdy F. (1978). Residual viruses in pork products. Appl. Environ. Microbiol..

[9] Petrini S., Feliziani F., Casciari C., Giammarioli M., Torresi C., De Mia G.M. (2019). Survival of African swine fever virus (ASFV) in various traditional Italian dry-cured meat products. Prev. Vet. Med..

[10] Stoian A.M.M., Zimmerman J., Ji J., Hefley T.J., Dee S., Diel D.G., Rowland R.R.R., Niederwerder M.C. (2019). Half-Life of African Swine Fever Virus in Shipped Feed. Emerg. Infect. Dis..

[11] Olesen A.S., Lohse L., Hansen M.F., Boklund A., Halasa T., Belsham G.J., Rasmussen T.B., Bøtner A., Bødker R. (2018). Infection of pigs with African swine fever virus via ingestion of stable flies (Stomoxys calcitrans). Transbound. Emerg. Dis..

[12] Karalyan Z., Avetisyan A., Avagyan H., Ghazaryan H., Vardanyan T., Manukyan A., Semerjyan A., Voskanyan H. (2019). Presence and survival of African swine fever virus in leeches. Vet. Microbiol..

[13] Mellor P.S., Kitching R.P., Wilkinson P.J. (1987). Mechanical transmission of capripox virus and African swine fever virus by Stomoxys calcitrans. Res. Vet. Sci..

[14] Focardi S., Morimando F., Capriotti S., Ahmed A., Genov P. (2015). Cooperation improves the access of wild boars (Sus scrofa) to food sources. Behav. Processes.

[15] Held S., Mendl M., Devereux C., Byrne R.W. (2000). Social tactics of pigs in a competitive foraging task: The ‘informed forager’ paradigm. Anim. Behav..

[16] Vial L. (2009). Biological and ecological characteristics of soft ticks (Ixodida: Argasidae) and their impact for predicting tick and associated disease distribution. Parasite.

[17] Rowlands R.J., Michaud V., Heath L., Hutchings G., Oura C., Vosloo W., Dwarka R., Onashvili T., Albina E., Dixon L.K. (2008). African Swine Fever Virus Isolate, Georgia, 2007. Emerg. Infect. Dis..

[18] Carrascosa A.L., Bustos M.J., de Leon P. (2011). Methods for growing and titrating African swine fever virus: Field and laboratory samples. Curr. Protoc. Cell Biol..

[19] King K., Chapman D., Argilaguet J.M., Fishbourne E., Hutet E., Cariolet R., Hutchings G., Oura C.A.L., Netherton C.L., Moffat K. (2011). Protection of European domestic pigs from virulent African isolates of African swine fever virus by experimental immunisation. Vaccine.

[20] Tignon M., Gallardo C., Iscaro C., Hutet E., Van der Stede Y., Kolbasov D., De Mia G.M., Le Potier M.-F., Bishop R.P., Arias M. (2011). Development and inter-laboratory validation study of an improved new real-time PCR assay with internal control for detection and laboratory diagnosis of African swine fever virus. J. Virol. Methods.

[21] Niederwerder M.C., Stoian A.M.M., Rowland R.R.R., Dritz S.S., Petrovan V., Constance L.A., Gebhardt J.T., Olcha M., Jones C.K., Woodworth J.C. (2019). Infectious Dose of African Swine Fever Virus When Consumed Naturally in Liquid or Feed. Emerg. Infect. Dis..

